# Impact of an Intensive Perinatal Handwashing Promotion Intervention on Maternal Handwashing Behavior in the Neonatal Period: Findings from a Randomized Controlled Trial in Rural Bangladesh

**DOI:** 10.1155/2017/6081470

**Published:** 2017-04-11

**Authors:** Pavani Kalluri Ram, Sharifa Nasreen, Kelly Kamm, Jelena Allen, Swapna Kumar, Mohammad Anisur Rahman, K. Zaman, Shams El Arifeen, Stephen P. Luby

**Affiliations:** ^1^University at Buffalo, Buffalo, NY, USA; ^2^icddr,b, Dhaka, Bangladesh; ^3^Stanford University, Stanford, CA, USA

## Abstract

One-quarter of neonatal deaths are attributed to infections. Maternal handwashing with soap may prevent neonatal sepsis. We examined impact of intensive handwashing promotion on handwashing behavior of mothers of neonates. In Matlab, Bangladesh, we randomly allocated pregnant women at 28–32 weeks' gestation to intensive handwashing promotion or control. Behavior change communicators used a participatory approach to motivate maternal handwashing with soap and provided soap and handwashing stations. In the neonatal period, we observed soap and water at handwashing places and, at the end of the neonatal period, we estimated impact on maternal handwashing by structured observation. Among 253 women enrolled, intervention households were between 5.7 and 15.2 times as likely as control households to have soap and water present at the handwashing station in the baby's sleeping area. Intervention mothers washed hands with soap 4.1 times as frequently as controls (95% CI 2.55–6.59); handwashing with soap at recommended times was infrequent in both intervention (9%) and control (2%) groups. Intensively promoting handwashing with soap resulted in increased availability of soap and water at handwashing places, but only a modest increase in maternal handwashing with soap. Novel approaches to motivating handwashing behavior to protect newborns should be developed and evaluated.

## 1. Introduction

In 2013, an estimated 2.8 million deaths occurred in the neonatal period globally [1]. Among these, one-quarter are attributed to infectious causes. Infections in the newborn period typically include pneumonia, sepsis, and those of the umbilical cord [1]. Deaths in the neonatal period have reduced more slowly than those in the postneonatal period since 2000. With the current complement and pace of public health efforts, neonatal sepsis is projected to continue to cause roughly 123,000 preventable deaths per 1000 live births in 2030 [1].

In an observational study of 23,662 infants in Nepal, Rhee and colleagues found that neonates of mothers who reported washing their hands were at 44% lower risk of mortality than neonates of mothers who did not report handwashing [2]. In the same region, Mullany and colleagues found that reported caregiver handwashing was associated with a 24% lower risk of umbilical cord infection [3]. Although these results are provocative, reported handwashing behavior consistently exaggerates observed handwashing behavior and so reported handwashing behavior is not a valid measure of handwashing [4–6]. Indeed, reported handwashing behavior is susceptible to social desirability bias, with wealthier or more educated respondents; that is, those whose children are already at lower risk of neonatal mortality, potentially more likely to overreport handwashing behavior than less wealthy or less educated counterparts [7, 8].

In a four-arm cluster-randomized controlled trial in Pakistan investigating the neonatal mortality effects of umbilical cord cleansing with chlorhexidine and handwashing with soap promotion separately and together, Soofi and colleagues found no benefit from promoting handwashing with soap [9]. The handwashing intervention tested in Pakistan consisted of provision of a bar of soap and “encouragement” by birth attendants for mothers and other family members to wash hands “before handling the newborn infant.” There was no assessment of handwashing behavior measures and thus, it is not clear whether the intervention did not affect handwashing behavior or whether improved handwashing behavior did not reduce neonatal mortality. Thus, there is a lack of published information regarding the efficacy of perinatal handwashing promotion interventions on maternal handwashing behavior in the neonatal period and on neonatal mortality.

Greenland and colleagues found maternal handwashing in the neonatal period in Indonesia to be infrequent, with handwashing occurring typically because of discomfort (e.g., due to sticky substances) or disgust-related motivators (such as apparent smell) [10]. In Bangladesh, Parveen and colleagues found that mothers perceived handwashing is an important approach to nurturing their newborns and young children and that they were more likely to practice the behavior when supported to do so by their families. Not having necessary materials for washing hands in close proximity was an important barrier to maternal handwashing in the neonatal period, particularly given prevalent social expectations regarding mothers and newborns staying almost exclusively in just 1-2 rooms or immediately outside the home [11, 12]. A barrier to maternal handwashing in rural Bangladesh was the lack of self-efficacy on the part of the mother to prioritize and carry out the behavior because of conflicting opinions from her in-laws, who may drive decision-making regarding childcare and household expenditures. Also, mothers felt that the neonatal period is a particularly busy time, with increased childcare responsibilities and sometimes increased housework, which interfere with her ability to wash hands even when she feels she should [13].

We developed an intensive intervention to improve maternal handwashing behavior during the neonatal period, addressing several of the key barriers and motivators identified by Parveen and colleagues, described above. Our objective, in a randomized controlled trial in rural Bangladesh, was to investigate the impact of this intervention on the handwashing behavior of mothers in the neonatal period.

## 2. Materials and Methods

This study was conducted in Matlab, a rural area 55 km southeast of Dhaka, Bangladesh. In 2007, 44% of mothers in the study area reported not receiving any antenatal care in the third trimester and 72% of births were attended by traditional birth attendants [14]. The neonatal mortality rate in this area was estimated at 29.9 deaths per 1000 live births [14]. The Health and Demographic Surveillance System (HDSS) in Matlab identifies pregnant women within two months of conception through bimonthly home visits. A Community Health Research Worker confirms a pregnancy in all women of child bearing age who have missed a menstrual cycle with a urine test during the bimonthly visit. Estimated delivery dates and contact information for each pregnant woman identified in the surveillance area are maintained by icddr,b Matlab field site staff. We recruited from 49 villages in the study area, which were not receiving any active maternal and child health or nutrition interventions.

Between October 2010 and October 2011, data collectors approached primiparous women identified in the study area through the HDSS pregnancy database with a due date between December 1, 2010, and December 1, 2011. We included primiparous mothers for the handwashing study because we anticipated that they would be at a uniquely teachable moment [15, 16]. Primiparous women were eligible if the following criteria were met:

- Woman planned to remain in the study area for at least one month before and one month after delivery.
- No other women living in the same household compound had previously taken part in either arm of the study.
- Women were not from household compounds that had participated in the qualitative research on motivators and barriers to handwashing in the neonatal period.
- Women were not enrolled in any other study; in some of the villages, a study on antenatal nutrition that enrolled women early in pregnancy was being implemented concurrently.

Eligible women were requested to provide signed informed consent. Prior to randomization, we recorded baseline knowledge of hygiene and beliefs pertaining to childcare and neonatal illness, perceptions of risk and severity of neonatal illness, antenatal care, and demographic information, using questionnaires and observations. Staff regularly contacted women during the two weeks preceding their estimated due date to identify births as soon as possible after delivery; women and their families were also encouraged to contact study staff for birth notification.

We used block randomization, using blocks of 4, to randomize participants to either the intervention or control arm. A study team member not involved in day-to-day field operations constructed the assignment table. The field team leader consulted the assignment table in order to determine the arm to which the participant was allocated. Data collectors were not blinded to the assignment status of participants, since the intervention included various hardware (handwashing stations and visual materials hung in the home), which were expected to be visible during the data collector's visit to the household.

### 2.1. Intervention

**Figure 1:**
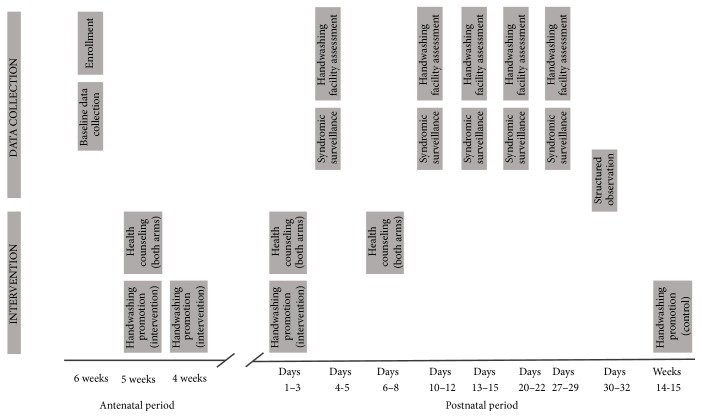
Timing of intervention and data collection visits.

We trained female behavior change communicators, who typically have Master's degrees and experience in data collection, to implement the intervention. The training consisted of didactic sessions, role plays, and field pilots and was delivered over 16 days. Both intervention and control participants received maternal and neonatal health counseling, patterned on information delivered in Projahnmo [17] (see visit schedule in Figure 1).

The behavior change communication strategy was based on a theoretical model constructed a priori that included concepts of the Health Belief Model and the heuristic model for teachable moments, described by McBride and colleagues [13, 15, 18]. As shown in a companion qualitative investigation, this model was used to understand drivers of maternal handwashing in the neonatal period [11]. Consistent with the findings from the qualitative work, behavior change communicators used an interactive approach to validate the prevalent perceived susceptibility of the neonatal period. They sought to have the mother and family members identify perceived barriers to their own handwashing, to address those barriers through behavioral solutions, and to enhance maternal self-efficacy for handwashing in the neonatal period. Study staff also improved access to handwashing materials in the physical environment to facilitate handwashing [18, 19]. Key constructs addressed in the handwashing intervention included the following:

- nurture as motivator for handwashing (to have healthy baby who grows well, mother's desire to take good care of baby)
- improved convenience (three handwashing stations were provided for continuous stocking with soap and water, including in the room where the baby would likely spend time; soap was replenished by study staff as needed throughout the perinatal period)
- cues to action (encouraged verbal reminders, provided cue cards with times for handwashing)

There is prior observational evidence supporting the beneficial effect of maternal handwashing before contact with the umbilical cord for prevention of neonatal infections [3]. Otherwise, we found no clear published evidence for times at which maternal or other caregiver handwashing may prevent pathogen transfer to/from hands in low-income settings. Therefore, we recommended the following times of* possible* pathogen transmission to the neonate for handwashing with soap: after respiratory secretion contact, before umbilical cord care, before breastfeeding, and after fecal contact. To stay consistent with typical handwashing promotion messages, we also recommended handwashing with soap before food preparation.

### 2.2. Outcome Measures

We used two principal methods to measure handwashing behavior: rapid assessments of the presence of handwashing materials and direct (structured) observations. At baseline and during postnatal days 4-5, 10–12, 13–15, and 20–22, the data collector identified the presence and location of a designated handwashing station(s), and the presence of water and soap at the handwashing station(s) (Figure 1). She assessed the presence of fully stocked handwashing stations anywhere in the home and specifically in the room where the newborn spent most of his/her time. Rapid assessments were unannounced.

The structured observation was conducted on days 30–32 postpartum and lasted 3 hours. During the observation period, a data collector positioned herself in the home/compound in order to assess handwashing behaviors at times of possible pathogen transmission to the neonate. Typically, the subject of observation was the mother; other family members interacting with the neonate (including touching, cooing, feeding, and bathing) were observed as well.

The primary outcome of interest was maternal handwashing with soap at times of possible pathogen transmission to the neonate. Secondary outcomes included the total number of times mothers were observed washing hands with soap during 3-hour observations; the maintenance of soap and water for handwashing anywhere in the home and specifically where newborns rest for most of the day; handwashing with soap by other household members at times of possible pathogen transmission; and the number of events observed among household members other than the mother washing hands with soap during the 3-hour observations.

### 2.3. Sample Size Estimation

When this study was being planned, we were not aware of data describing the observed frequency of hand cleansing before cord cleansing. Substantially more information is available regarding the frequency of hand cleansing after defecation. Thus, to estimate the required sample size for measuring the impact of handwashing promotion on maternal handwashing behavior, we assumed that the probability of observed handwashing with soap after a fecal contact event would be 0.20 in the control arm [20] and that the probability would increase to 0.40 in the intervention arm. We also assumed one fecal contact event to be detected per 3-hour structured observation. Based on these assumptions, 80% power, and a .05 significance level, we estimated that we would need to conduct 3-hour structured observations among 80 respondents in each study arm. To account for clustering of behavior at the individual level (i.e., an individual is more likely to behave like herself than she is to behave like others), we introduced a design effect of 2, increasing the sample size up to 160 in each arm. We further increased the desired sample to 200 per arm to account for potential loss to follow-up, and adverse perinatal outcomes such as maternal or neonatal death, since structured observations would not be feasible or appropriate in such cases.

### 2.4. Data Analysis

The primary outcome of interest was based on structured observation data and reflects the proportion of intervention-recommended times at which one or both hands were washed with soap. In order to compare the frequency of handwashing during the observation period between treatment arms, we used mixed linear regression to calculate the difference in the mean number of observed handwashing events between arms, separately among and other household members, in an intent-to-treat analysis. Although not prespecified, we also generated rate ratios to compare the proportion of intervention-recommended times at which hands were washed in the two study arms; we used log binomial regression, accounting for repeated measures at the caregiver level to adjust standard errors. We assessed differences between the study arms, with respect to observed handwashing with soap among other family members interacting with the neonate. In supplemental multivariate analyses, we adjusted for baseline differences between study arms. We also report the proportion of mothers who maintained soap and water at at least one handwashing station in the home and at a handwashing station in the room where the neonate was cared for and compare the intervention and control groups at each time point that this outcome was observed.

This trial was registered at http://www.clinicaltrials.gov (Identifier: NCT01309321). This study was reviewed and approved by the Research and Ethical Review Committees at icddr,b: International Centre for Diarrhoeal Diseases Research, Bangladesh (PR-10036).

## 3. Results

**Figure 2:**
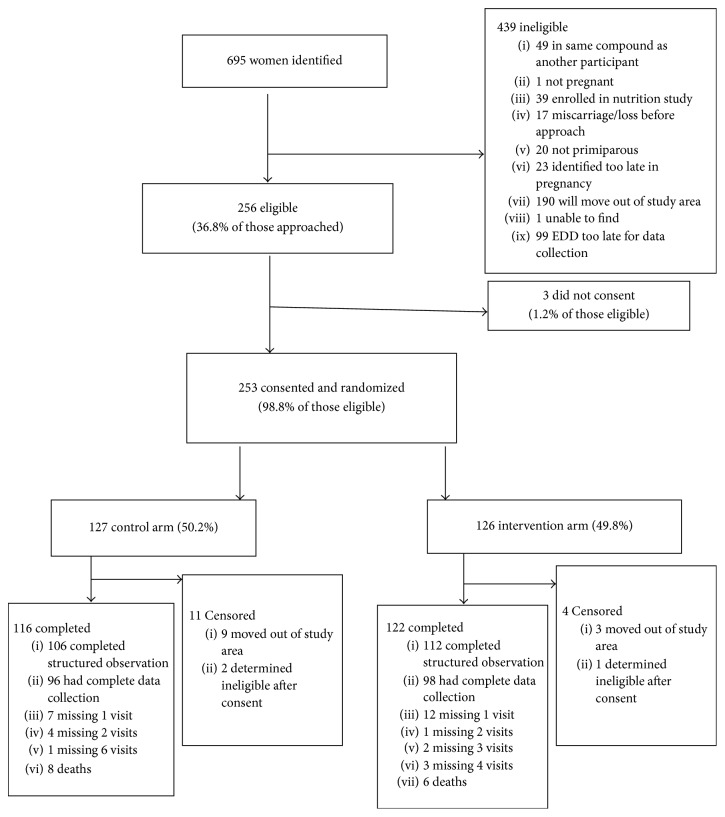
Eligibility, allocation, and completion of data collection among pregnant women, Matlab, Bangladesh, 2010-11.

Between October 2010 and October 2011, we identified 695 pregnant women reported to be between 28 and 32 weeks of gestation in the study villages (Figure 2). Common reasons for ineligibility included identification too late in the pregnancy or a due date too late for complete data collection (24%), previous enrollment of another pregnant woman in the same compound (11%), and enrollment in another study (6%). Of the 256 (37%) women who met eligibility criteria, 253 (99%) consented to take part. We randomized 126 (49.8%) participants to the intervention arm and 127 (50.2%) to the control arm. One participant assigned to the intervention arm and two in the control arm were later found to be ineligible because another woman residing in the same household compound had previously been enrolled in the study; data collection was discontinued upon identification of the ineligibility criteria. Therefore, we analyzed data from 125 women in each arm.

Participants in the two treatment arms had similar baseline measures, including demographics, soap and water at a handwashing station, wealth measures, and number of years of education for the mother (Table 1). The arms were similar with respect to roof and wall materials. Whereas the number of years of education for the participant herself was similar in the two arms, participants in the control arm reported a mean of 7.2 years (SD 3.3) of education for the husband, compared to those in the intervention arm who reported a mean of 8.8 years (SD 12.0). Access to a deep tubewell was also somewhat different between the two groups (59% controls, 68% intervention).

At baseline, the majority in each arm, 67% of controls and 60% of intervention participants, indicated that their main handwashing station was near the tubewell. None of the participants in the control arm and only 2 (2%) in the intervention arm were observed to have their main handwashing station inside the house. Almost all participants were found to have water, but just 15 (12%) in each arm were observed to have bar soap at the main handwashing station; other types of soap were rarely observed.

We documented 2 (2%) stillbirths and 4 (3%) neonatal deaths in the intervention arm and 6 (5%) stillbirths and 2 (6%) neonatal deaths in the control arm. There were no maternal deaths. All births were notified either by the respondent/family or upon telephone call by study staff following the estimated date of delivery.

**Figure 3:**
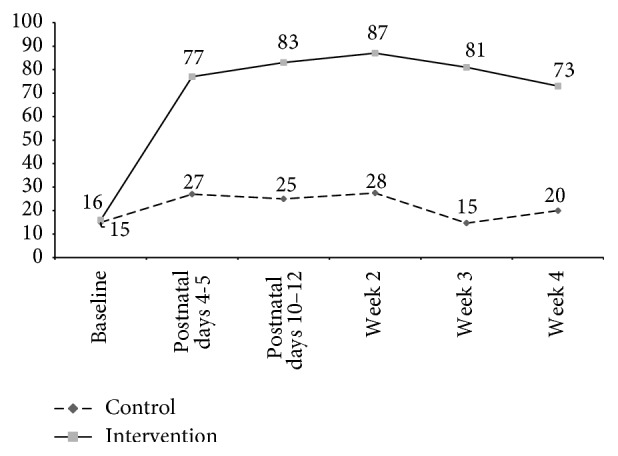
Proportion of households with at least one handwashing station with soap and water, by treatment arm, Matlab, Bangladesh, 2010-11.

During rapid assessments conducted on postnatal days 4-5, the proportion of households observed to have soap and water at a handwashing place was 77% in the intervention arm and 27% in the control arm (RR 3.0, 95% CI 2.11–4.15, *p* < .0001) (Figure 3). At all five assessments made during the neonatal period, households in the intervention arm were 3 or more times as likely as households in the control arm to have at least one handwashing station observed to have soap and water present. All five assessments were successfully completed in 104 intervention households and 97 control households. Among these, 39% of intervention households and 3% of control households were observed to have soap and water at a handwashing station at all of the visits.

**Figure 4:**
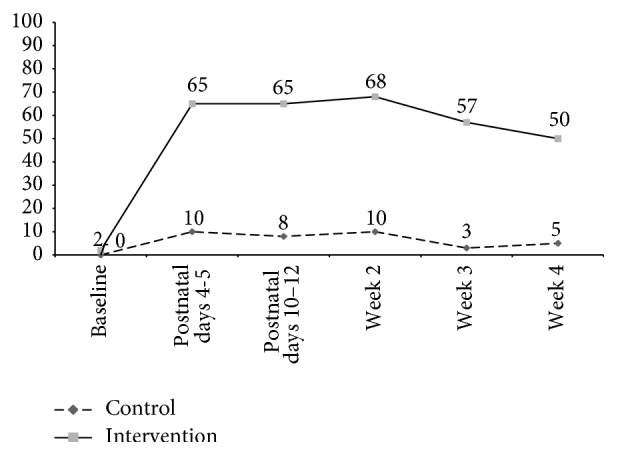
Proportion of households with a handwashing station with soap and water observed in the baby's sleeping area, by treatment arm, Matlab, Bangladesh, 2010-11.

The majority (84%) of respondents in the intervention arm and 44% of respondents in the control arm reported that the primary handwashing station used by the respondent was located in the baby's sleeping area, at postnatal days 4-5 (RR = 2.2, 95% CI 1.72–2.92, *p* < .0001), decaying to 51% of respondents in the intervention arm and 9% of respondents in the control arm during the last week of the neonatal period (RR = 4.7, 95% CI 2.53–8.76, *p* < .0001). Soap and water were observed at the handwashing station in the baby's sleeping area at 65% of intervention households and 10% of control households at the observation conducted at postnatal days 4-5 (RR = 5.6, 95% CI 3.12–10.14, *p* < .0001) (Figure 4). Across the five visits made in the neonatal period, intervention households were between 5.7 and 15.2 times as likely as control households to have soap and water present at the handwashing station in the baby's sleeping area.

During days 30–32, we completed 3-hour structured observations of 106 (85%) control and 112 (90%) intervention participants (Table 2). Loss to follow-up is described in Figure 2. Whereas the frequency of handwashing* with water alone* was similar in the two groups (mean 1.6 events in intervention arm, 1.5 events in control arm), mothers in the intervention arm washed their hands* with soap* with a mean of 0.82 times (SD 1.2) during the 3-hour observation period, compared to 0.20 times by mothers in the control arm (RD 0.61, 95% CI = 0.37–0.86) (Table 2). During the 3-hour structured observations, 45% in the intervention group, compared to 17% in the control group, were observed to wash hands with soap and water at least once. Mothers in the intervention arm were 3.9 times as likely as those in the control arm to be observed washing their hands at the times recommended as part of the handwashing intervention (95% CI 1.23–2.02). The risk differences and risk ratios were not substantively altered in multivariate analyses accounting for baseline differences between the groups with respect to mother's education and water source. Handwashing with soap was more frequently observed among intervention mothers than controls before breastfeeding (RD 0.65, 95% CI 0.03–0.99) and after fecal contact (RD 0.10, 95% CI 0.04–0.17) (Table 3). Overall, the prevalence of handwashing with soap at both these times was low, even in the intervention group (8% before breastfeeding and 14% after fecal contact). There were no significant differences between the two groups in handwashing with water alone at either of these times. No umbilical cord care events were observed, given that observations were conducted after the typical time of cord separation. None of the respiratory secretion contact events in either group were followed by handwashing with soap.

Handwashing with soap was significantly more frequently observed in intervention arm household members overall and at the recommended times; however, handwashing with soap was infrequent in household members in both arms (handwashing at 6% of recommended times in intervention arm and 1% of recommended times in the control arm).

At baseline, 23% of control (29/124) and 20% of intervention (25/125) participants said that another person reminded them to wash hands on a typical day. At follow-up, 28% of control arm (30/106) and 36% (40/110) of intervention said that someone reminded them. At baseline, 40% of control (50/124) and 46% of intervention (57/125) participants said they reminded someone else to wash hands on a typical day. At follow-up, 44% (47/106) in the control arm and 70% in the intervention arm (77/110) reported doing so.

## 4. Discussion

Intensive handwashing promotion employing emotional drivers, improving convenience, and providing visual cues led to only a modest increase in the frequency of handwashing with soap among new mothers in rural Bangladesh during the first 30 days after the birth of their child. The intervention resulted in greater maintenance of handwashing materials in locations where mothers are cocooned with neonate. However, the overall proportion of events accompanied by handwashing was low in the intervention arm compared with the times that we recommended for handwashing. Handwashing with soap was significantly higher before breastfeeding and after fecal contact among intervention mothers than among controls; however, washing with soap after fecal contact was less common than the prevalence of this behavior observed among mothers of young children in Bangladesh and elsewhere, underscoring the substantial barriers to handwashing faced by mothers of neonates [21].

McBride et al. has described that pregnancy is a transformative moment in a woman's life, at which time she is reenvisioning her role of herself as well as creating and reacting to the expectancies of her pregnancy outcome (i.e., the new baby she will care for) [15]. Curtis and colleagues have found that mothers and other caregivers commonly report that they wash hands out of a wish to nurture their young children [22]. Despite employing nurture as a primary driver of handwashing behavior and including in the intervention all secondary caregivers, not only the mother, there was limited behavioral impact of the intervention.

We sought to increase the convenience of handwashing by providing soap and handwashing stations and encouraged maintenance of soap and water at handwashing locations throughout the newborn period. Handwashing station placement was guided by the mother and other relatives such that the mother would have access to soap and water in the key locations where she expected to spend time with the neonate. Still, the finding that only 39% of households in the intervention were found to have soap and water at a handwashing place at all five of the rapid observations suggests that maintaining handwashing materials at fixed locations at all times was difficult in the newborn period, that such maintenance had not become habitual, or that this approach to handwashing was not acceptable to our study population. Hand cleansing technology that does not necessitate as frequent replenishment may be needed to further improve hand hygiene in the newborn period.

While the intervention increased the frequency of handwashing, mothers and others washed hands with soap at less than 10% of the times we recommended for handwashing. Since we completed structured observations at the end of the neonatal period during postnatal days 30–32, we cannot know whether handwashing was more common or less common during the early days of the newborn period, when babies may have been perceived to be particularly vulnerable but mothers may have been particularly fatigued or getting adjusted to the new household routine following the birth.

Mothers in several low-income countries have indicated that their responsibilities increase in the postnatal period, often without increased support for household or child care activities from others [12]. We recommended handwashing after respiratory secretion contact, before umbilical cord care, before breastfeeding, after fecal contact, and before food preparation. Observational findings of the benefit of washing hands before contact with the umbilical stump [3] and the available evidence that a number of Gram positive organisms that colonize the nasopharynx and Gram negative organisms found in stool are commonly associated with sepsis in neonates and young infants [23, 24] drove the decision-making regarding recommendation of handwashing after respiratory secretion contact, before umbilical cord care, before breastfeeding, and after fecal contact. Handwashing with food preparation was added in order to stay consistent with typical handwashing promotion efforts in Bangladesh and to facilitate the development of maternal handwashing behavior relevant to postneonatal morbidity. For busy new mothers to comply with handwashing at all the recommended times may have been extremely difficult.

Increasing evidence regarding the role of healthy intestinal, skin, and mucosal microbiota affirms that most organisms transmitted from mothers to neonates are not pathogenic. Indeed, transfer of many commensal organisms from mother to neonate represents an important element of developing healthy microbiota and immune systems [25–28]. Therefore, recommendations regarding the optimal times to interrupt pathogen transmission, while preserving the transfer of healthy microbiota to neonates, should be articulated based on an increased understanding of the mechanisms of transmission of pathogenic and commensal organisms from mothers to neonates.

Hands get recontaminated after handwashing quickly in heavily contaminated household environments such as rural Bangladesh [29] and, thus, infrequent handwashing may contribute to pathogen transmission to neonates. We do not yet understand how much handwashing of mothers and others needs to be increased, and at which times of potential pathogen transmission, in order to reduce the risk of infection in newborns. There may also be a role for hand cleansing options that are waterless or that can confer residual bacteriostatic benefit to improve the convenience and thereby the frequency of hand cleansing, or the effects of even infrequent hand cleansing.

We restricted our study to primiparous mothers, since they might have been more susceptible to behavior change given their relatively larger change in self-definition, compared to multiparous mothers. However, this sample may have decreased the representativeness of our study; mothers with a newborn who also have infants or older children to care for may be less likely to change handwashing behavior because of time constraints, or because their current pregnancy does not lead to a dramatic alteration of their self-definition or role in the family and society since they already have children. We used observed handwashing as our primary outcome of interest. Persons being observed by a stranger, such as a study data collector, may alter their behavior compared to usual practice, potentially increasing their handwashing in the presence of the observer [29, 30]. Such reactivity may be exacerbated by the provision of handwashing materials and promotion of handwashing behavior in the intervention arm. Our observations took place at the end of the neonatal period. Handwashing behavior may have been different earlier in the newborn period, when threat perceptions may have been higher but household routines may have been disrupted by the arrival of the new baby. Lastly, we did not perform a formal process evaluation, a process that can elucidate whether the intervention was not implemented as designed or whether the implementation proceeded as planned but the intervention was not efficacious in changing behavior as intended.

### 4.1. Conclusion

We found that a handwashing promotion strategy that consisted of both providing the necessary materials for handwashing and intensive behavior change communication resulted in anemic improvements in handwashing behavior among mothers of newborns. While mothers in the intervention arm were significantly more likely to maintain needed handwashing materials in the places where they spent time with their newborns, and more frequently washed their hands, handwashing with soap was observed at a minority of the times we recommended. Promotion of handwashing with soap before umbilical cord care should be emphasized in antenatal and neonatal care messaging, given existing evidence supporting prevention of umbilical cord infections by caregiver handwashing. Understanding the extent to which hand hygiene behavior of mothers and other caregivers needs to improve at other times of potential pathogen transmission would support the development of feasible guidance on handwashing to reduce neonatal infections. Novel and feasible approaches to motivating handwashing behavior change among mothers and other caregivers to protect newborns should be developed and evaluated and, if effective, their impacts on neonatal health should be investigated.

## Figures and Tables

**Table 1:** Comparison of participants in the control and intervention arms at baseline, pregnant women at 28–32 weeks' gestation, Matlab, Bangladesh, 2010-11.

Characteristic	Control group (*N* = 125)	Intervention group (*N* = 125)
*Demographics*		
Mother's mean age (SD)	20.3 (2.4)	19.9 (2.3)
Mean number of people in household (SD)	5.7 (2.4)	6.0 (2.9)
Mean number of prenatal visits (SD)	0.7 (0.5)	0.79 (0.4)
Health care worker talked about handwashing with soap (%)	5 (4.0)	8 (6.4)
Reported knowing about clean delivery kit (%)	3 (2.4)	4 (3.2)
*Water and sanitation*		
Main source of drinking water: Tubewell (%)	117 (97.2)	121 (94.4)
Location of main handwashing station (%)		
Near surface water	21 (16.9)	27 (21.6)
Near tubewell	83 (66.9)	75 (60)
Other	20 (16.1)	23 (18.6)
Materials present at main handwashing station (%)		
Water	122 (98.4)	122 (97.6)
Bar soap	15 (12.1)	15 (12.0)
Powdered soap	6 (4.8)	4 (3.2)
Liquid soap	0 (0)	1 (0.8)
Ash	14 (11.3)	8 (6.4)
Water and soap together	18 (14.5)	20 (16.0)
*Wealth and education*		
Possession of a working television (%)	50 (40.3)	59 (47.2)
Availability of electricity/solar panels (%)	87 (70.1)	87 (70.0)
Tin roof (%)	122 (98.4)	119 (95.2)
Tin walls (%)	115 (92.7)	112 (89.6)
Mean years of education for mother	7.5 (2.3)	7.60 (2.5)
Mean years of education for husband	7.2 (3.3)	8.82 (12.0)

**Table 2:** Handwashing behavior as measured by structured observations, by treatment arm, Matlab, Bangladesh, 2010-11.

	Control group	Intervention group	Risk difference	Adjusted risk difference^1^	Risk ratio (95% CI)	Adjusted risk ratio^1^ (95% CI)
# observations completed	106	112			n/a	

*Mothers*						
Mean number of handwashing events (with or without soap)	1.7 (SD 1.6)	2.5 (SD 1.9)	0.80 (0.32, 1.27)	0.82 (0.35, 1.29)	1.48 (1.22–1.79)	1.49 (1.24, 1.81)
Mean number of events of handwashing with soap	0.20 (SD 0.52)	0.81 (SD 1.2)	0.61 (0.37, 0.86)	0.62 (0.37, 0.87)	4.10 (2.55–6.59)	4.06 (2.53, 6.54)
Handwashing with soap at recommended times^2^	2.3% (18/776)	9.1% (78/854)	0.07 (0.04, 0.10)	No convergence	3.94 (2.09, 7.44)	3.86 (2.05, 7.27)

*Other household members* ^3^						
# observations completed	105	111			n/a	
Mean number of events of handwashing with soap	0.06 (SD 0.23)	0.32 (SD 0.93)	0.26 (0.07, 0.44)	0.26 (0.08, 0.45)	5.52 (2.32, 13.12)	5.46 (2.30, 13.00)
Handwashing with soap at recommended times	1.2% (4/343)	6.1% (23/379)	0.05 (0.02, 0.08)	No convergence	5.20 (1.80, 15.09)	5.58 (1.94, 16.11)^4^

^1^Adjusted for mother's education and water source; referent = control.

^2^Recommended times for handwashing: after respiratory secretion contact, before umbilical cord care, before breastfeeding, after fecal contact, and before food preparation.

^3^In one household in each arm, only the mother was observed during the SO.

^4^Log Poisson model used in place of log binomial model due to lack of convergence of model. Log Poisson given consistent but less efficient estimates of RR compared to log binomial models.

**Table 3:** Handwashing behavior before breastfeeding and after fecal contact, as measured by structured observations, by treatment arm, Matlab, Bangladesh, 2010-11.

	Control group	Intervention group	Risk difference	Adjusted risk difference^1, 2^	Risk ratio (95% CI)	Adjusted risk ratio^1, 2^ (95% CI)
*Before breastfeeding*						
Number of events observed	396	410				
HWWS	6 (2)	31 (8)	0.0649 (0.0307, 0.992)	*∗*	5.11 (1.89, 13.85)	5.18 (1.91, 14.00)
HWW	16 (4)	26 (6)	0.0276 (−0.0050, 0.0601)	0.0278 (−0.0049, 0.0605)	1.67 (0.91, 3.08)	1.67 (0.90, 3.09)
No HW	374 (94)	353 (86)	Referent	Referent	Referent	Referent

*After fecal contact*						
Observed events	219	242				
HWWS	10 (5)	33 (14)	0.1027 (0.0401, 0.1652)	*∗*	2.99 (1.42, 6.30)	3.21 (1.53, 6.77)
HWW	25 (11)	28 (12)	0.0144 (−0.0547, 0.0834)	0.0081 (−0.0613, 0.0775)	1.12 (0.65, 1.92)	1.17 (0.67, 2.04)
No HW	184 (84)	181 (75)	Referent	Referent	Referent	Referent

HWWS: handwashing with soap.

HWW: handwashing with water.

HW: handwashing.

^1^Comparison of HWWS versus no HW.

^2^Adjusted for mother's education (if included water source, no convergence).

^*∗*^No convergence using log binomial or Poisson model.
